# An Appeal from Bethlehem
*Poems by a Prisoner in Bethlehem. Edited by J. Percival, Esq. London: Effingham Wilson.


**Published:** 1851-04-01

**Authors:** 


### Art. VI.—AN APPEAL FROM BETHLEHEM.*

Mr. J. Percival, the Editor of this little volume of poems, is a kind and benevolent man, with his *heart* in its right place. He has his hobby, and, like many of us, may at times be disposed to ride it *a little* too hard; nevertheless, we greatly commend him for his undeviating and zealous assiduity in pursuit of what he conceives to be an object worthy of the devotion of his life. Mr. Percival describes himself as "*Hon. Sec. to the Alleged Lunatics' Friend Society,*" an association organized for the detection and liberation of persons unjustly confined in asylums, or elsewhere, on the ground of insanity. Such a society would be entitled to our warmest approbation, and would deserve both public and private patronage and support, if it could be satisfactorily established

* Poems by a Prisoner in Bethlehem. Edited by J. Percival, Esq. London: Effingham Wilson.

that there was a necessity for its existence. It is not, however, our intention on this occasion to argue the question with Mr. Percival.

The author of these Poems is *Mr. Pearce.* Of this unfortunate youth, the Editor observes—

"He is a man of gentlemanly birth and education, being related to the family of the late Dr. Pearce, Bishop of Rochester, and having been educated in Paris for the medical profession, is now subject to the common fare and diet of the hospital; and he has scarcely the means of procuring those little additional articles of luxury, or that style of dress suited to his station in society, which the liberality of the rules of the asylum would allow to him, and the absence of which must render his position so much more painful and humiliating. Of a property of about £600, about £80 alone remains to him, the rest having been consumed chiefly by the law expenses of his committee in endeavouring to recover it, and in those of a commission de Lunatico Inquirendo on his case, procured when he thought that he was of sound mind, and that he ought to have been restored to society. In consequence of the violence which he attempted, about ten years ago, against the person of his wife, in a fit of apparently groundless jealousy, when his reason was obscured by manifest delusions, she and her relations have entirely abandoned him, and have cut off all communication with him; and his own friends, who resided in Paris, were involved in the ruin brought upon so many by the late French Revolution, and are now no longer able to assist him."—Preface, p. viii.

Mr. Percival, influenced by feelings which do him much honour, undertook the extremely hazardous speculation of printing, at his own cost and risk, this volume of poems, composed by Mr. Pearce during his confinement in Bethlehem Hospital as a criminal lunatic.

In speaking of this public institution, Mr. Percival remarks—

"A great improvement has taken place in the management of the patients there, and I have heard it well spoken of, both in society, and by patients who have been confined there; but there are also, occasionally, some exceptions, and the general appearance of the building will convince any sensible man that much prejudice is yet to be overcome, and much improvement still to be made there. I never visit my friend in this asylum without being oppressed, on my approach to it, by the gloomy exterior of the building, and wounded by the severity of the interior, to a degree that makes it painful to return there, and requires of me considerable resolution to do so. The windows are obscured by thick iron bars, which we know now are no longer necessary, as security can be combined with lightness and elegance, and the only defence that I have heard for them is the insufficient excuse of their enabling the keepers to give the wards more thorough ventilation, without fear of escape, or injury to the patients. The walls of the wards and the cells are of bare brick, whitewashed, without any pretence to comfort, ornament, or protection from violence. The cells are dungeons lighted by a small window at the top, inaccessible to the patient, so that a patient confined to his bed from week to week, has no sight to cheer him, but on all sides a rough cold blank, on which his excited and deluded imagination may imprint any ideas that his native propensities may incline him to, terrible or sensual, extravagant or revolting, without any correction or any distraction. The plea for having thus the bare whitewashed walls is, that of cleanliness, of freedom from vermin and from infection; but they rather betoken a niggardness* of charity—for true charity would provide becoming comforts for the patients, and render it compatible with cleanliness and healthiness by proper service around them. Lastly, the yards, which are the only places in which the patients can walk for exercise, are small and cheerless, and partake of the severity of the building. Perhaps the best thing to be done with Bethlehem would be that Government should purchase it, as a house of correction, or convert it into a National Gallery; and that the hospital should be removed further into the country, and placed in an open and airy situation, with large grounds around it."—Preface, p. xi.

There is, we regret to say, some justification for these observations; but, perhaps, the features complained of by Mr. Percival, are inseparable from a large public asylum like Bethlehem Hospital. It has been the constant aim of the medical officers and governor to remove, as much as possible, the prison appearance and character of the institution; but, without re-building the asylum, it would be impossible to make it a cheerful, and at the same time a safe place of residence for the kind of patients transferred to its wards.

We must confess that, on entering the portion of the asylum apportioned to criminal lunatics, we have been always much impressed with the truly prison-like character of all the arrangements. It should never be forgotten, that parties sent to this hospital, after being acquitted on the plea of insanity, are so exonerated because they are the *victims of disease,* which disease has deprived them of the healthy and right exercise of their mental faculties; and although, in a strictly legal sense, they are *prisoners,* they are entitled to as much commiseration, sympathy, and attention as the other patients in the asylum; and every indulgence, consistent with their safe custody, ought to be allowed to them. Upon the treatment of the poor criminal lunatic, Mr. Percival eloquently and feelingly observes—

"More severity could not be exercised, consistently with humanity, to the most criminal and responsible, who have, humanly speaking, no excuse. Equity, therefore, requires that some difference should be made between them and those whom justice does not consider amenable to her on account of some mental infirmity. Many persons in society,

* "I would prefer supposing a want of judgment in desiring rather to extend the charity to numbers, than to deal faithfully to those who are recipients of it."

I know, alarmed at the numerous instances of acquittal of parties, upon the plea of insanity, after offences of a serious character against the person, and the person even of the most exalted members of the state, are hurried by their fears into an undue appreciation of the benefit that the criminal may derive from such a repair from the consequences of his outrages. But, if they would visit the asylum as I have done—if they would enter, after the door has been unlocked to them, the stone passage leading to the criminal wards, on two sides railed in with a grating of iron bars, an inch square, behind which, as though they were wild beasts in cages, the maniacs are confined, crawling, jabbering, shouting, or taking their hurried and excited exercise—if they could hear the echo of the signal given by the key of the servants along the grating in front of them, and see their wan and haggard friend descend the stone steps opposite with the keepers, with whom they have to converse through the bars of his prison-house, on the most private subjects, unless they are admitted as a favour into the comparative privacy of the keepers' little chamber—if they would afterwards reflect that within these bare walls, behind these harsh and heavy gratings, in hearing of these sounds, in sight of this wretchedness, the miserable object whom they visit has to drag on his weary existence, in society perhaps unsuited, perhaps degrading to him, from day to day, from month to month, from year to year, and so on in dull and never-ending monotony, they would soon feel that death, if it could be met with propriety, were preferable to such a reprieve, and transportation to the colonies infinitely preferable to such indulgence. But if, in addition to these considerations of the personal and physical privations and annoyances of the patients so confined, any man will reflect a moment upon the necessary and constant separation of them from the charms and solace and delight of female society, they will acknowledge that no fate can be more terrible than theirs; no doom more melancholy; no disaster so fraught with calamity and apprehension to the soul and spirit, as well as to the body—than such isolation from all the guides, all the encouragements, all the aids to cultivating a happy, cheerful, and resigned disposition—all that soothes the spirit, and gives energy to the soul, in her combat between virtuous and vicious propensities."—Preface, p. xiii.

The poems before us are of a higher order than many it has been our duty, and perhaps misfortune, to read, written by persons *supposed* to be in the possession of their right mind, and who have luxuriated in the privilege of residing *outside* the walls of a lunatic asylum. But, perhaps, we have a right to expect such a result. It has been maintained, by no mean authority, that all truly good poets should be mad; that insanity is one of the most important elements in the poetic character. We will not argue the question, but leave Tennyson, Marston, Rogers, and other eminent living poets, to settle the matter between them. But to the poems. Our space will not admit of any extended quotation. The subjoined sonnet will commend itself to the judgment and taste of our readers.

"SONNET.

"Come, my Camilla, for the spangled morn,
Is up, and, smiling, greets the milk-white thorn;
And you and me, let 's hasten to obey
The thousand heralds of the genial May.
List, from this open casement to the birds
Chiming their matin carols; and the herds,
See how they browse the breakfast of the plain,
While the white sheep in companies remain.
The daisy, buttercup, and pale primrose
Gem the green fields, and modestly disclose
Their beauties. And the bolder-cultur'd flow'rs
Fill the soft air with perfume from the bow'rs.
Come, then, Camilla, sin no more by staying,
But, hand in hand, come let us go a-Maying."—p. 71.

The sonnet entitled "An Appeal for Little Children," is also deserving of our warm commendation—

"SONNET.

"AN APPEAL FOR LITTLE CHILDREN.

"Look down, just Heaven, on the little child,
  The gentle symbol of man's future power;
Whether in city, or the country wild,
  O, guard each rising, lisping human flower.
Ten thousand curses on the recreant hand
  That persecutes the tiny sinless race,
That dares to cloud, as with a wizard's wand,
  The fair spring morning of an infant's face.
Philanthropists, go on, nor heed the storm:
One Lord his *Shafts* doth *bury** in the form
  Of giant tyranny—his boast to rule,
  The guide and patron of 'The Ragged School.'
When Death, the master, stands beside his bed,
*That man* shall bow in blissful dreams his head."—p. 59.

The little volume is worth purchasing, independently of the benevolent purpose for which it is published. We sincerely trust the worthy Editor will be enabled, by the sale of the volume, to create a fund for the comfort and support of Mr. Pearce during his melancholy incarceration in Bethlehem Hospital.

"* An allusion to the title of Lord Ashley's father, the Earl of Shaftesbury.—ED.

